# Whole-Genome Sequence of *Staphylococcus epidermidis* Tü3298

**DOI:** 10.1128/genomeA.00112-16

**Published:** 2016-03-10

**Authors:** Josephine C. Moran, Malcolm J. Horsburgh

**Affiliations:** Institute of Integrative Biology, University of Liverpool, Liverpool, United Kingdom

## Abstract

*Staphylococcus epidermidis* Tü3298 is a frequently used laboratory strain, known for its production of epidermin and absence of the *icaABCD* operon. We report the whole-genome sequence of this strain, a 2.5-kb genome containing 2,332 genes.

## GENOME ANNOUNCEMENT

*Staphylococcus epidermidis* is a frequent cause of hospital-acquired infections, exhibiting increasing levels of antimicrobial resistance that are a growing concern. The bacterium primarily causes infections of catheters and other inserted medical devices. The life cycle of *S. epidermidis* as a normal and dominant resident of the human skin flora affords this opportunistic pathogen with access to these inserted medical devices that is difficult to prevent.

As a colonizing species of human skin, *S. epidermidis* must compete with other bacteria for space and nutrients (1, 2). Many staphylococci produce lantibiotics, antibiotic-like peptides, as a competition mechanism (3). *S. epidermidis* Tü3298 produces the lantibiotic epidermin, active against many Gram-positive bacteria (4). This was the first lantibiotic shown to be post-translationally modified, and remains one of the best-characterized staphylococcal lantibiotics, with its biosynthesis, regulation, mode of action, and the nature of bacterial resistance all having been elucidated (3). The optimized protocol for transformation by electroporation of *S. epidermidis* Tü3298 (5) made this strain ideal for use in studies of gene function via mutagenesis. Subsequently, *S. epidermidis* Tü3298 has been used in studies of the role of *agr* (6–8), σ^B^ (9), and SepA (10). This strain is also widely used as an *icaABCD* negative control in biofilm assays (11, 12).

Libraries were prepared for sequencing with Nextera DNA kits (Illumina) and were sequenced on the Illumina MiSeq platform. Contigs were assembled using VelvetOptimiser (Velvet version 1.2.06) and were annotated using Prokka (13). Sequences were assembled into 162 contigs; the average coverage for the assembly was 14.

The *S. epidermidis* Tü3298 genome is 2,459,658 bp. It contains 2,332 protein coding sequences, 360 of these are hypothetical.

The genome sequence of *S. epidermidis* Tü3298 will contribute to easier genetic manipulation of this strain, and will enable further sequence-dependent studies in the future.

### Nucleotide sequence accession numbers.

This draft genome sequence has been deposited in the ENA under the accession no. CZRO00000000. The version described in this paper is the first version, CZRO02000000.
